# Preschool Teachers’ Beliefs towards Children with Autism Spectrum Disorder (ASD) in Yemen

**DOI:** 10.3390/children7100170

**Published:** 2020-10-06

**Authors:** Sahar Mohammed Taresh, Nor Aniza Ahmad, Samsilah Roslan, Aini Marina Ma’rof

**Affiliations:** 1 Department of Foundation of Education, Faculty of Educational Studies, Universiti Putra Malaysia (UPM), Serdang 43400, Malaysia; sahartaresh30@gmail.com (S.M.T.); samsilah@upm.edu.my (S.R.); ainimarina@upm.edu.my (A.M.M.); 2 Department of Kindergarten, Faculty of Education, Taiz University, Taiz, Yemen

**Keywords:** autism spectrum disorder (ASD), preschool teachers, religious belief, social beliefs, personal beliefs, Yemen

## Abstract

It is perplexing that some preschool teachers not only advise parents who have children with autism spectrum disorder (ASD) to go to religious healers, but also attribute such neurological disorders to the curse of the “evil eye” or vaccines. Although it is now the twentieth century, this behavior simply reflects the concerns of over-protective teachers and the cultural misperceptions about the actual definition of ASD. In Yemen, the term “ASD”, with its wide range of symptoms, is still ambiguous among preschool teachers. Thus, in a rather insightful piece for the education community, this study has attempted to look beneath the surface of the beliefs (religious belief–social belief–personal belief) of Yemeni preschool teachers regarding ASD. Based on the data collected from 213 teachers (20–30\31–40-~≥40 age) in the Taiz district, this study found that misconceptions specific to autism spectrum disorder were strongly evidenced among teachers who taught preschoolers. Due to personal ignorance and growing superstitions, these teachers tend to believe the society’s perceptions of ASD, thus resulting in the ignorance of scientific views. However, the mass media can increase this group’s awareness of ASD by continually assessing the inaccurate views on ASD, and correcting them. And by influencing the teachers to take a more conceptual scientific approach in serving their special needs students, furthermore, by informing preschool teachers of children’s rights in normal life in the future through providing children with an optimal chance of development by early intervention.

## 1. Introduction

The Republic of Yemen’s decision in 1991 was the first legislation for the care and rehabilitation of children with disabilities in Yemen. In 1999, the Higher Committee for the Care of Handicapped Rehabilitation was established, which launched the Handicapped Care and Rehabilitation Fund. Unfortunately, the Handicapped Care and Rehabilitation Fund considered autism spectrum disorder (ASD) as one of the disability categories. In their strategy for the years 2004–2018, the first goal was to change society’s view towards children with disabilities by raising the level of awareness of their rights and capabilities. During that period, Yemen was rapidly improving towards urbanization, while suffering a deteriorating economy. According to The United Nations Human Development Report ranked in 2007, Yemen was estimated to be one of the poorest countries in the world. Specifically, it was rated number 140 out of 182 (United Nations Development Program-Human Development Report 2009). Low admission to education became an essential poverty-related issue in Yemen. According to the World Bank, 87% of the poor in Yemen are illiterate or have not completed primary school. All of this was reflected in the individuals’ beliefs and attitudes towards children with disabilities in general and children with developmental disorders, as is the case with children with ASD. Thus, these beliefs can be used to explain any strange phenomenon, including ASD. Due to a lack of visible symptoms in children with ASD, many may attribute ASD with stigma and misconception. For example, uninformed people may perceive a child with ASD to be someone who looks “normal” but acts voluntarily in ways that violate social norms [1,2]. In a longitudinal study of families with children diagnosed with autism, it was confirmed that autism spectrum disorder is often characterized by pervasive impairments in social interactions, communication skills, and restricted patterns of behaviors and interests [1]. The severity of the symptoms varied significantly; children with ASD often have intellectual abilities within normal borders [3]. Despite their ordinary intelligent abilities, preschoolers with ASD show a prorate weakness in language, social skills, and executive performance, thereby making it tough for them to learn and to forge relationships with their peers. Consequently, these limited capabilities contribute to an increased misunderstanding regarding this disorder, and such misunderstanding is associated with different positive and negative beliefs [4,5].

In recent years, families and communities have been reported to be the primary cause of ASD misunderstanding [6]. Armstrong and Fitzgerald [5] also confirmed that different cultures often use explanations and observations with significant differences in descriptions and classifications to describe the disability. To depict cultural differences, the Multicultural Disability Advocacy Association (MDAA) of NSW, Australia, suggested that while the disability is often explained as a medical concept in Western cultures, other cultures view it as a punishment or gift from a higher power [7].

The above perceptions strongly affect the behavior of disabled children as well as their guardians [8]. Considering the misconception that the disability is related to specific actions, there is a high risk that families and communities deny children diagnosed with autism their rights to receive adequate help [6]. When explanations for the disability are placed outside appropriate interventions, there is also a high risk that the incapacity of the disabled children will be kept private [9].

Previous studies have found that knowledge of ASD is associated with a lower rate of stigma [10,11,12]. However, this is not always the case because most ASD information that the public receives comes from the media, which is made up of stereotypical beliefs or convictions that people see as truth [13]. However, these beliefs are not backed by sufficient proof [14]. There are many beliefs about autism spectrum disorder that originate from extreme superstition and cultural views. Hence, several investigations have been carried out to get an in-depth explanation regarding the root of the problem. The results of such investigations point toward a pattern of negative opinions towards autism spectrum disorder, whether as a punishment for the family’s previous sins, the mother’s negligence, or the work of wicked ghosts [15,16]. Similar to an investigation into South Korean society, the psychiatrists were more likely to deduce that “bad” mothers and pregnant mothers who were depressed and introverted caused their child to become autistic [17]. On the other hand, another study determined how five Muslim parents who had children diagnosed with autism interpreted their religious beliefs to empower them to seek an understanding of their children’s special needs [18]. Other published studies support the above works, proving that the beliefs that the family is at fault for causing a children’s mental disorder are extremely spiritual and religious [10].

The effect of spirituality is amazingly profound among some believing families. Parents or guardians were able to self-heal by accepting that Allah, the Creator of the Universe, had chosen them to be the caretakers of children diagnosed with autism due to their piety, diligence, nobility, and capacity to support. This notion of Allah’s involvement does play a role in enabling the affected families to reach a more profound acceptance of family members with disabilities. In other words, a child with ASD brings them closer to their religion and the Creator. It was also found that Pakistani and Bangladeshi Muslim believers learned that not only did Allah put an autistic child in their care as a result of destiny, but also because Allah wished to test their family and to see if they would continue to be excellent or unkind to the child. Beyond this, however, it seems that teachers, in general, are still plagued by misconceptions. For instance, one previous study on various cultural groups examined the role of culture in influencing teachers’ beliefs within an ecological framework—discussing the nature and causes of childhood disability and the teachers’ ideas about treatment. Another study revealed that the teachers held both biomedical and socio-cultural views that reflect duality in their beliefs [19]. The view of both parents and teachers is a relevant factor in the above relationship. With their consent, some researchers sought to determine the cognitive, emotional, and developmental characteristics of children with the disorder by assessing the parents’ and teachers’ beliefs and knowledge about specific areas of the disorder. The views of the parents and teachers were not interrelated nor accurate, which made cooperation between the two parties difficult [20].

People with less experience and knowledge of ASD often hold inaccurate beliefs about the disorder [21]. Based on the discussion above, a lack of knowledge regarding autism spectrum disorder creates a wide avenue for social stigma, which means that children diagnosed with autism and their families may be more stigmatized than other children with disabilities. In a study in which adults and teens with ASD were asked about their challenges, the respondents reported feelings of being evaluated and rejected by their families and friends. Some even stated that they were victims of bullying at social events [22]. Besides, another study evaluated the knowledge of 15 early childhood preservation teachers about ASD adaptation as an entry questionnaire to autism spectrum disorder. Of the 15 elements with true/false reaction options, the results illustrated that pre-service child teachers did not know the subject matter.

Furthermore, they had some misunderstanding about ASD etiology and the behaviors of children with ASD. Regarding etiology, it was found that 93% of preschool teachers did not identify ASD as a developmental disorder, while 60% of them believed that the children could “overcome” their situation. Only 53.3% of them recognized the genetic contribution to the disorder, and 20% of them wrongly indicated the effect of trauma as a cause for the visible behaviors of ASD.

Moreover, 73.3% of pre-service teachers thought that behavioral treatment was not an effective intervention, and 66.7% of them affirmed that children with ASD were entirely similar to others. The results also indicated that 46.7% of them did not identify with the justification for early interventions to help children with ASD, and 26.7% of the participants considered it an erroneous behavioral intervention for a child with ASD. This finding is similar to the results of Barned et al. [23], which indicated that the pre-service preparation for ASD in preschool is inadequate [24].

Another noteworthy study reviewed the beliefs regarding ASD among the general public, including teachers in the United States and Canada (*n* = 823), as well as people dealing with childcare services in the State of Idaho. The results showed that nearly all participants properly understood the genetic and neurological cause of ASD (not parenting, drugs, or a recent diet) that can be recognized early on in a child’s life for prompt intervention. The study also emphasized the correct community knowledge of ASD to facilitate early identification and effective intervention. The results suggest professional development courses for childcare providers as well as effective channels for transmitting accurate information such as broadcast and online media from which the general public, especially followers of ethnic minority groups, are most likely to learn about ASD [25].

Theoretically, through the health belief model (HBM), it can explain the preschool teacher’s beliefs based on this theory, as this theory describes health-related behaviors and medical decision-making. The HBM was developed initially in the 1950s to explain why people did not participate in preventive disease programs [26,27]. Preschool teachers’ beliefs can be a barrier to healthy behavior toward their children, and preschool teachers’ belief in their actions can protect their children in the class and estimate when to take action [28].

Interestingly, this data presented a whole collection of findings that differed from those reported in the Eastern world, specifically the Arab world. Hence, it is critical to correct such misunderstanding and stigma surrounding ASD, mostly in countries where ASD services are scarce. ASD in Arab countries such as Yemen has received relatively little attention from the research community so far.

Preschool teachers’ judgments of the perceived barriers and the perceived benefits of action define the course of action taken; these two components together form the dimension outcome expectations [28]. This includes preschool teachers’ “perceived costs involved in seeking a diagnosis (e.g., time, don’t have evidence, social stigma, how to voice their concern to parents, not knowing who to contact, refuse the parents, etc.).

A current analysis of ASD research in Arab countries revealed a total of 75 articles being published between 1992 and 2012 [29]. In contrast, the United States has been producing international ASD research articles for years, including 1040 publications in 2010 alone [30]. Thus, there is an urgent need for further research, as in the case of the current study, to assess teachers’ beliefs about ASD in Yemen.

### 1.1. Religious Belief

Beliefs and behavioral health are rooted in religion and spirituality. Many religious and spiritual traditions observed in cultures around the world are associated with health practices [31]. Aside from having a profound influence on the views of health and diseases in many cultures, these two elements can also have implications for the field of health communication [32]. In essence, religion affects various social, cultural, and personal aspects, such as traditional norms, values, and customs [33]. Consequently, traditional cultural perspectives inevitably influence ASD treatment recommendations. One important aspect to consider when discussing ASD from a cultural perspective is that there could be many weird explanations. While ASD is clearly defined by its visible features in all cultures, the systems of cultural and religious belief vary drastically and, therefore, create a different experience of illness with different religious perspectives.

Among Muslim families, it is generally recognized that Allah puts an autistic child under their care not only because of fate or reincarnation, but also because Allah wants to test that particular family to see if they can care for the child. This concept, quite simply, prohibits any inhumane treatment or immoral behavior towards children with ASD [10]. Some families, on the other hand, embrace the child’s disabilities as qadar/kismat (fate) [34], a situation which is similar to that reported by other Muslim families in other studies, especially because religious considerations have long been taken at the family level. For example, Muslim mothers in Turkey mentioned Allah, fate, spells, and evil spirits as the causes for having disabled children, while strong traditional beliefs exist concerning the possibility of a child’s restoration, which in turn determines their actions relating to help or treatment [35]. In a small part of the Indian subcontinent, various cultural and ritual superstitions surround the disabled. Some Punjabi Muslims believe that treating people who are either called defective or crazy, will ensure their direct admission to heaven when they die. Some also blame evil crimes for the health deterioration of children [4].

Besides, Pakistani and Bangladeshi Muslims believe that Allah wants the parents of children diagnosed with autism to play a pivotal role in improving their children’s life skills through various forms of therapy. The Muslim community here trusts that a suitable therapy is one that improves both the health and the soul [36]. However, some traditional families become angry at the Western point of view on ASD treatments and exclude their children diagnosed with autism from a proper diagnosis [10]. For instance, parents would refuse to work with certified professionals, focusing on the child’s weaknesses instead of highlighting the child’s potential [10]. This calls for a form of willingness to find religious healers who ignore the negative side and focus on the positive side of ASD instead [10]. As part of their spiritual belief system [4], Pakistani and Bangladeshi Muslims have also resorted to prayers and pilgrimage to seek guidance in helping their children diagnosed with autism reach their maximum potential and mission in life [17].

Religious implications on beliefs concerning children with developmental problems are not only limited to Muslims believers [10] but also include other religious groups. As reported in a previous study, 55% of Latina mothers believe that their autistic child is a sign of Allah’s existence [37], while other Latina mothers believe them to be blessings or gifts from Allah that would give them the chance to do good and to surrender parts of their lives to serve others [38]. Furthermore, it was found that Latin Americans have the option of opting for non-traditional treatments, such as the use of folk healers [39].

Meanwhile, numerous Hindu parents of children with “mental disorders” in the United States believe that Allah has given them the child as a response to sins committed in their previous lives [40]. While white Americans use traditional treatments and professional services [39], many African-Americans would seek advice from friends, families or church members before going to professionals. Asians, on the other hand, are often hesitant to seek professional assistance; they are more likely to “go alone” and get help if they cannot manage their child [38,39].

In contrast, it was found that ultra-orthodox Jewish families often change community dynamics by receiving medical advice from a Rabbi [41]. If a given treatment modality is contradictory, then the advice of the Rabbi is followed [10,42] regardless of whether insights into the disability are deemed as pessimistic or a celebration of life. The underlying concept, however, rests on the fact that religion often plays an important role in understanding the ASD experience [10,43,44], and therefore, families who believe that ASD can be cured tend to follow the mandate of the treatment designed to cure, while unbelieving families are less likely to challenge the course of the disorder [45].

The above discussion highlights the importance of religion. Based on the available knowledge on culture, religion can help families emotionally and socially, while playing an important role in boosting the capacities of parents to care for their child. However, religion may be misinterpreted, thus affecting the way families make decisions about further treatment and help.

### 1.2. Social Belief

Culture affects an individual’s common belief system and serves as an explanatory model for disorders such as ASD [31]. In spite of the contrasting interests with which ASD has to contend, multiculturalists assessed the pressures of ASD differently, and these evaluations offer both negative and positive reviews [31]. On the negative side, culture makes people believe that ASD is a stigma. A stigma is known as the manifestation of a diverse form [46]. It is discriminating in nature and does not correspond to the normative expectations of society, and therefore results in a deteriorating social identity for affected individuals or even groups. Stereotyping against those with disabilities often occurs because of the negative perception of ASD [47]. The stigma surrounding ASD has resulted in discrimination against not only children diagnosed with autism but also their families [48]. It is often negatively perceived that children with ASD would find it hard to achieve success in life. Some parents go to the devastating extent of hiding their children diagnosed with autism from the community to prevent the family from losing respect [31].

In other communities, the disabled group is pushed to social exclusion and often their quality of life is put at risk [31]. Discrimination against children with ASD has been broadly described in the United States and around the globe to acknowledge the burden that stigma can bring to both parents and children with ASD [49].

Because the reason for ASD is still unknown, numerous multicultural groups have propped up their own belief about the reasons for developmental disorders. When people’s beliefs about ASD etiology were reviewed, it was found that people, including preschool teachers, believed that genetic, environmental factors, and birth-related events were the contributing factors. Despite having been debunked in multiple extensive studies, anecdotes from parents support the suggestion that vaccines can cause developmental disorders in children. Millions of parents firmly believe that genetics, birth trauma, illness, inheritance, perinatal damage, the environment, or some combination of these disorders could cause children to become autistic [50,51]. However, many parents also believe that genetic factors are the main cause of these disorders [52,53]. Given the vast storehouse of myths and half-truths, more scientific inquiries on various cultural groups and their genetic and biological make-up will help confirm the causes of ASD. Cultures have often perpetuated false beliefs in the field of ASD because different groups may have a specific way of explaining atypical behaviors and beliefs [54].

### 1.3. Personal Belief

Beliefs inevitably take on a more influential role than knowledge in determining how people organize and identify tasks and problems. Moreover, beliefs are the strongest indicators of people’s behavior [55]. Preschool teachers often stigmatize children with ASD, highlighting individual perceptions that may be incorrectly ascribed to people with disabilities. For some people, the regularity of the education system may be the most important factor in determining their beliefs [33].

In a study among special administrative region university students living in Macau, most of the participating students confirmed that the ASD etiology stemmed from negligent and emotional parents. However, only about one third of the students believed in genetic etiology. There was also a significant difference in the strength of each belief. On average, the participants expressed mild-to-moderate agreement with statements describing paternity as the etiology for ASD. Instead, they responded with a slightly neutral opinion or reaction to statements regarding genetic factors as the etiology of ASD [54]. Furthermore, a study by Hoekstra et al. proved that the personal beliefs of preschool teachers influences the transference of information to the parents of an ASD child [56].

In Arab countries, Hasnain et al. (2011) highlighted the commonly used Arabic word for ASD (التوحد), as individuals with a behavioral, mental, physical, and/or emotional disability. However, for many, the word itself is commonly interpreted as “to introvert” or “withdraw”. Therefore, many individuals may have incorrectly interpreted the nature of ASD as introversion [31]. In China, a lack of ASD knowledge has been confirmed among preschool teachers. Similarly, this lack of knowledge has been reported due to the literal translation of both Chinese terms for autism spectrum disorder —zibizheng Gūdān—as “loneliness” or “introvert disease”, which implies a more misunderstand form of psychological etiology [54].

The aforementioned findings provide support for the positive belief that children with ASD are talented in art, music, language, and computing [21]. However, figuratively, it is believed that children with or without ASD may possess extraordinary abilities. Moreover, their sophisticated skills may have been confused with general interests in public, social, and scientific discourses. Thus, having in-depth knowledge about ASD is equally important for members of the society, including preschool teachers. Countering misconceptions is best done through professional training that focuses on equipping teachers with the skills they need to improve the experience of children with ASD [33].

Needless to say, researchers have pointed out the importance of conventional directions—what preschool teachers have traditionally believed, their religious, social, and personal views on ASD, as well the relationship between several independent variables such as their education level and teaching experience and one dependent variable, which is a preschool teacher’s belief about autism spectrum disorder. Therefore this study aims to answer the following two questions:

- What are the beliefs of preschool teachers concerning children with autism spectrum disorder?
- What are the potential differences in the beliefs of preschool teachers towards ASD according to their age, education level, and teaching experience?

## 2. Methodology

### 2.1. Research Design

A quantitative descriptive survey research design was utilized in this study to assess the different forms of beliefs of preschool teachers in general education towards ASD in Yemen. This design has been used in some previous studies such those of Qi et al. and Chirico et al. [54,57] to assess belief about ASD among teachers and parents as well.

### 2.2. Instrument

Overall, the instrument used in this study, specifically the ASD Beliefs Questionnaire (ABQ), consisted of two sections. The items in the developed instrument were adapted from questionnaires by Harrison et al. [58] and Al-Sharbati et al. [59], with their permission. The first section focused on the demographic information of the respondents (i.e., gender, age, marital status, education level, and teaching experience). The second section consisted of 20 ABQ items to assess the religious (five items), social (seven items), and personal (eight items) beliefs of preschool teachers towards ASD. For this section, the respondents were required to provide their responses according to a five-point Likert scale with the endpoints of “ from strongly disagree” (1) to “strongly agree” (5). In addition, a “less agree” option was included in the response scale, instead of “neither agree nor disagree,” in order to avoid respondents trying to guess their responses.

Firstly, the religious belief, in this case, represented one’s belief on how ASD and behavioral health disorders are rooted in religion and spirituality. Examples of religious belief include “ASD is likely a result of a curse or evil eye put upon or inflicted on the family” or “I think it is possible to treat ASD by consulting with religious therapists”. Secondly, the social belief, in this case, represented one’s common belief based on the cultural effect as an explanatory model system for ASD. Examples of social beliefs include “My society thinks that vaccination causes ASD” or “ASD holds a social stigma in some communities, such as Yemen society”. Lastly, the personal belief, in this case, represented one’s perception (maybe correct or incorrect) on children with ASD. Examples of personal beliefs include, “I think the majority of children with ASD suffer from mental retardation” or “I believe ASD can develop due to child maltreatment”.

For this study, the developed instrument was back-to-back translated carefully. The equivalence of the translation was first reviewed by a panel of five experts (special educational needs teachers). Meanwhile, the face validity of the instrument was initially verified by four education specialists (two psychologists and two psychiatrists) who were professionally trained in the field of special education. This group of professors rated the clarity and appropriateness of the Likert scale statements. Necessary adjustments, including the rewording of certain phrases, were made according to the group’s observations and suggestions. Following the adjustments, the percentage of the agreement from the group achieved 0.94. The face validity of the Yemeni version of the Likert scale statements was subsequently confirmed during the pilot study that involved 45 preschool teachers. All items were revealed to be easily understood, and no changes in wording were needed. The responses during the pilot study were not included in the actual study. Besides, the internal consistency of the instrument recorded Cronbach’s alpha coefficient of more than 0.83, which reaffirmed the reliability of the items.

### 2.3. Procedure

The school administrators in one of the biggest cities in Yemen, specifically the Taiz district, were contacted to obtain their permission to conduct this study that involved their preschool teachers. After obtaining the permission of school administrators to conduct this study, preschool teachers were randomly selected to complete a self-administered ABQ on their beliefs towards ASD. The data collection was conducted during the summer of 2018. A total of 250 preschool teachers were randomly selected to complete a questionnaire on their beliefs towards ASD. All questionnaire sets were then returned to the researcher. However, 37 returned questionnaire sets had missing information. The exclusion of these incomplete questionnaire sets resulted in a final sample of 213 preschool teachers.

### 2.4. Data Analysis

The categorization of scores in this study represented the different levels of beliefs of preschool teachers, specifically religious, social, and personal beliefs, towards ASD. The recorded scores were interpreted according to three distinct levels: (1) low level of accurate beliefs (mean value ≥ 3.34); (2) moderate level of accurate beliefs (mean value of between 1.67 and 3.33); (3) high level of accurate beliefs (mean value ≤ 1.66) [60]. In other words, a low level of accurate belief implies that the preschool teacher displays disbelief towards ASD; a moderate level of accurate belief implies that the preschool teacher displays some misbelief towards ASD; a high level of accurate belief implies that the preschool teacher displays an accurate belief towards ASD.

Accordingly, the obtained survey responses were analyzed using IBM SPSS (version 19). First, the demographic information of the participating preschool teachers as survey respondents was descriptively analyzed. Second, the reliability and validity of ABQ were determined based on the results of Cronbach’s alpha coefficient. Third, after ensuring that the obtained data were normally distributed, multilinear regression was carried out to see the differences among dependent variables (religious, social, and personal). Two-way ANOVA was carried out to assess the relationship between independent variables (i.e., age, education level, and teaching experience) and the dependent variable (personal belief). Besides this, post-hoc analysis (Scheffé post-hoc criterion for significance) was also performed.

On the other hand, data for the other dependent variables (religious belief and social belief) reported non-normal distribution. Moreover, the homogeneity equality of variance was less than 0.05 (*p* = 0.042). Therefore, the non-parametric test—the Kruskal–Wallis test (K–W test)—was valid and used instead of the ANOVA test. Thus, it was concluded that assumptions for the two-way ANOVA had been violated. The transformation was rather difficult because the specimens were small and difficult to interpret, especially in the two-way ANOVA. Therefore, the non-parametric test (K–W test) was valid and applied for religious belief and social belief [60].

## 3. Results

The respondents of this study were preschool teachers in normal schools in Taiz. Only 5% of the participants have reported that they have had previous contact with children with ASD, which was expressed through the (Yes, No) open question “Have you ever contacted a child with autism?”. Table 1 presents the sample distribution according to gender, age, education level, and teaching experience.

### 3.1. Preschool Teacher’s Belief Regarding Autism Spectrum Disorder

The first research question focused on determining the beliefs of preschool teachers towards ASD. With respect to this research question, the descriptive statistics (i.e., mean, standard deviation, and weight mean) of the categorical data in this study were acquired. And multilinear regression to see the differences lie between dependent variables (religious, social, and personal).

#### 3.1.1. Descriptive Analysis

Table 2 shows the weight means for each belief among the preschool teachers according to three distinct levels for interpretation. In particular, the religious belief regarding preschool teachers’ belief towards ASD was found to be at a moderate level, whereas their social and personal belief of preschool teacher’s belief towards ASD was located at a low level. In other words, preschool teachers display inaccurate beliefs towards ASD resulting from the religious, social, and personal beliefs of the preschool teachers.

#### 3.1.2. Results of Multi Linear Regression

Multiple Linear Regression analysis is an appropriate statistical method that allows us to examine and understand how the relationship among the independent variables are related to the dependent variable and to explore the forms of these relationships (between two or more variables of interest). Regression analysis is also used to understand which among the independent variables are related to the dependent variable, and to explore the forms of these relationships.

One-way ANOVA was employed to determine the effects of the dependent factor on the two independent factors as shown in Table 3. The F statistic was 9.880, indicating that the independent’s variables (social belief, personal belief) are statistically significant. The results showed that the two independent factors (social belief, personal belief) have an effect on the dependent factor (religious belief) (*p*-values ˂ 0.000) as illustrated in the same Table 3. This reflects that the alternative hypothesis of 3 variables are accepted, and that both social and personal beliefs were associated with religious belief.

The structural model assessment, as shown in Table 4, provides an indication of the hypotheses tests or question research of this current study. If hypothesis H1 (β = 0.142, t = 4.234, *p* = 0.000) and H2 (β = 0.089, t = 2.121, *p* = 0.035) are accepted, these could be the correlation relationships between Social Belief and Personal Belief empathy which significantly predicts that the Religion Belief dependent variable holds a strongly positive correlation relationship which is highly significant at the 0.01 level (2-tailed).

The second research question was to assess the potential differences in the beliefs of preschool teachers towards ASD according to their age, education level, and teaching experience as shown below.

### 3.2. The Interactiont of Age, Education Levels, and Teaching Experience on Religious Believe

Based on Table 5; The Kruskal–Wallis Test (K–W test) interaction effect results indicated that there was a significant difference in religious belief between age (20–30 and 31–40 and above 40 years) and education levels (Diploma and Bachelor) (*p* = 0.023; *p* = 0.017). Moreover, there was not a significant difference in preschool teachers’ belief regarding religious belief between age and education level (high school) (*p* = 0.115). The mean score for the age between 20–30 was less than the mean score for the age between 31–40 years. Thus, it was concluded that the age between 20–30 years and above 40 years had lesser inaccurate beliefs than the age between 31–40 years.

Moreover, the results indicated that there was a significant difference in preschool teachers’ beliefs based on religious belief between ages (above 40 years) and teaching experience (above 10 years) (*p* = 0.001). There was not a significant difference in preschool teachers’ belief regarding religious belief towards ASD between age and teaching experience (from 5 years and below and between 5–10 years) (*p* = 0.238; *p* = 0.226). This is shown in Table 6 below.

Based on the K–W test, there is a significant difference between the mean scoring of teaching experiences (from 5 and below and between 5–10 years) and education levels (*p* = 0.023; *p* = 0.000) on preschool teachers’ beliefs based on religious belief. However, there is no significantly different between teaching experiences (above 10 years) and education levels (*p* = 0.920) on preschool teachers’ beliefs based on religious belief towards ASD, as it follows in Table 7.

### 3.3. The Interaction Effect of Age, Education Levels, and Teaching Experiences on Social Belief

The K–W test interaction effect results indicated that there was a significant difference in preschool teachers’ beliefs regarding social belief between ages and education levels (diploma) (*p* = 0.006). Moreover, there was not a significant difference in preschool teachers’ beliefs regarding social belief between age and education levels (high school and Bachelor) (*p* = 0.130; *p* = 0.116). The mean score for the age between 20–30 was less than the mean score for the age between 31–40 years. Thus, it was concluded that the age between 20–30 years and above 40 years had a lesser effect on preschool teachers’ beliefs based on social belief than the age between 31–40 years, as is shown in Table 8 below.

The results also indicated that there was a significant effect of social belief on preschool teachers’ belief between age and teaching experience (between 5–10 years and above 10 years) (*p* = 0.002; *p* = 0.002). There was not a significant effect of social belief on preschool teachers’ beliefs between age and teaching experiences (from 5 years) (*p* = 0.542). See Table 9

According to the K–W test, there is a significant difference between the mean soring of teaching experiences (between 5–10 years above 10 years) and education levels (*p* = 0.000; *p* = 0.006) on preschool teachers’ beliefs based on social belief. However, there is no significant difference between teaching experience (from 5 years and below) and education levels (*p* = 0.237) on preschool teachers’ beliefs based on social beliefs. See Table 10

### 3.4. The Interaction Effect of Age, Education Levels, and Teaching Experiences on Personal Belief

Two-way ANOVA was employed to determine the effects of age, education levels, and teaching experience on personal beliefs. An assumption of two-way ANOVA was checked, and the normality test was also checked for each factor. Each factor showed either that it was normally distributed or slightly skewed positively, which was in the accepted range of skewness. Homogeneity equality of variance was checked using Levene’s test and it was found that the equality of variance assumption was met (*p* = 0.215). Thus, it can be concluded that it had not violated the homogeneity of variance assumption for 2-way ANOVA.

As seen in Table 11, the age effect (age between 20–30, age between 31–40, and age above 40 years) and working experience have no significant effect on preschool teachers’ beliefs based on personal belief (*p* = 0.192; *p* = 0.859). Only the effect of education levels has a significant effect on the scores of personal belief (*p* = 0.002).

However, when a two-way interaction effect between each study factor was checked, it was found that there was a significant interaction effect between age factors with education levels and teaching experience on the scores relating to personal belief (*p* = 0.033; *p* = 0.018). It indicated that the effect of the age effect on personal beliefs may differ between education levels and teaching experiences. The results also found that there is no significant effect between education levels and teaching experience (*p* = 0.637).

Regarding the result of the post-hoc Scheffe’s test, and based on the personal beliefs of preschool teachers (aged 31–40), there is a significant effect (mean = 2.57; *p* = 0.002) on preschool teachers’ beliefs towards ASD. In addition, the personal beliefs of preschool teachers with a high school degree have significant differences (mean = 6.39; *p* = 0.000) on preschool teachers’ beliefs towards ASD. Furthermore, the personal beliefs of preschool teachers who have teaching experience below 5 years have a significant effect on (mean = 2.02; *p* = 0.021) preschool teachers’ belief towards children with ASD.

## 4. Discussion

The current study aimed to gain a better understanding of the impact of religious, social, and personal beliefs on one’s perception of etiology, symptoms, signs, and socio-demography correlating to ASD. Overall, the survey conducted in this study revealed that many preschool teachers had misconceptions about ASD. They also repeatedly associated ASD with religion and believed that religious healers could treat children with an autism spectrum disorder. Some also believed that traditional therapy might be useful, as ASD was an atonement for previous sins [61]. Family members or relatives have sought help from traditional healers and shamans, including Rabbis [62], in part due to the lack of a known cure for ASD. Another reason why most parents struggle with finding the right help for ASD is that they believe that ASD is a scourge from Allah and that the disorder is a result of the curse of the evil eye. Neni et al. [63] validated this in their research which demonstrated that 18.2% from their sample of members of the public still believed that evil spirits caused the disability. These findings indicate that religion in many types of culture and practices is known to serve as an explanatory system to provide a reason for the causes or treatment of ASD.

Religious beliefs of preschool teachers aged above 40 years with teaching experience above 10 years have a significant effect on preschool teachers’ beliefs towards ASD. In other words, the religious beliefs of those aged above 40 with teaching experience above 10 years were the most significant. Their religious beliefs caused inaccurate beliefs toward children with ASD. The reason for that is that older generations (who have more education and more teaching experience) were raised in an era that had strong religious/traditional interpretations of health. These findings indicate that in Yemen, religion plays a major role in identifying the causes and treatment of ASD. Yemen is among the Muslim countries that belief traditional therapy to be useful for treating cases such as ASD. The most common traditional therapy applied is Quran therapy.

On the other hand, the religious beliefs of preschool teachers with an (high school) education level and teaching experiences (below 5 years and between 5–10 years) have no significant effect on their belief toward children with ASD. Based on their religious beliefs, these preschool teachers had accurate beliefs about ASD. This result could be explained by the fact that preschool teachers with low levels of education attempt to seek more knowledge through attending workshops and training programs that can help them deal with children who have problematic behavior instead of relying only on their religious interpretations.

This study revealed many common social assumptions on ASD among preschool teachers. Their contradicting views are debatable. Some teachers with many years of experience had more accurate beliefs on ASD than those with fewer years of teaching. In addition, more experienced teachers did not consider or view vaccination as a cause of ASD when compared to less-experienced teachers. Many of them believed that ASD was a genetic disorder. Mitchell et al. [25] found that most preschool teachers stayed up-to-date through social media, and yet, they still had inaccurate information about ASD; for instance, the assumption that ASD might be linked to vaccines. Most preschool teachers with less than five years of teaching experience claimed that children with ASD are indeed geniuses with distinguished skills, confirming the results reported by Khanna et al. [64] The preschool teachers in the study also believed that children with ASD were susceptible to negative social consequences and general stigma. Similarly, Al Sharbati et al. [59] pointed out that children with disability challenges in Oman were once hidden within the familial household and had limited access to educational or remedial services.

The social beliefs of preschool teachers aged 31–40 with an educational level (diploma) as well as teaching experience (between 5–10 years and above 10 years) have a significant effect on the accurate belief of preschool teachers towards children with ASD. In other words, the social beliefs of preschool teachers aged 31–40 with educational levels (diploma) as well as teaching experience (between 5–10 years and above 10 years) were the most significant. Their social beliefs caused inaccurate beliefs toward children with ASD. This might be due to preschool teachers with more than 10 years of teaching experience being uninterested in updating their knowledge compared to those who have less than 5 years of teaching experience. Moreover, low-experienced preschool teachers tend to update their knowledge to improve their careers by attending more training programs [2]. Furthermore, the Yemeni culture has social restrictions that are reflected in the preschool teachers’ interpretations of any phenomenon such as ASD. For instance, some preschool teachers consider ASD to be a stigma, which is very common in Yemeni society [59]. These inaccurate beliefs held by many Yemeni people might shape the preschool teachers’ cultural beliefs affecting their career.

On the contrary, based on social beliefs, the Age effect (20–30; 31–40; >40) with the education levels (high school–Bachelor) as well as teaching experience (below 5 years) have no effect on the accurate belief of preschool teachers towards children with ASD. This may be due to those preschool teachers with less than 5 years of teaching experience, and a high school education being more concerned about gaining basic teaching and behavior modification skills [23]. It may be the case that preschool teachers who have a high education level (Bachelor) may have received university courses in special education needs.

The preschool teachers had personal assumptions or perceptions on ASD that they had formed through thoughts and experiences. This study found that some teachers thought cell phones were a reason for the emergence of ASD and that most children with ASD were introverts. Such findings confirm the results of John et al. [21] in that those preschool teachers with less experience might find it difficult to distinguish between ASD and introversion, thus thinking that they are similar. Another common personal belief was that an autistic child is suffering from “loneliness”—an error also prevalent among preschool teachers in China [33]. The Chinese teachers’ inaccurate response to ASD may partially stem from the Chinese terms for ASD that translates to “loneliness disease” or “isolation disease’”, implying a more psychological etiology. Some Arabic terms like ((التوحد translated as “loneliness”, explains why some of the preschool teachers in this study equated this disorder with the feeling of being lonely. However, the response rates indicate that this disorder could be cured, so training in early childhood development would be adequate to provide teachers with a knowledge base in neurodevelopmental disorders, as recommended by Liu et al. [33]

The personal beliefs of preschool teachers aged (31–40) with an educational level (high school) as well as teaching experience (below 5 years) have a significant effect on the accurate belief of preschool teachers towards children with ASD. In other words, the personal beliefs of preschool teachers aged (31–40) with an educational level (high school) as well as teaching experience (below 5 years) were the most significant. Their personal beliefs caused inaccurate beliefs toward children with ASD. This means that preschool teachers with high teaching experience can change their thinking towards ASD better than preschool teachers with teaching experiences for less than 5 years. On the other hand, the personal beliefs of preschool teachers aged 20–30 (>40) with an education level (Diploma and Bachelor) have no significant effect on their belief towards children with ASD. This indicates that they have accurate beliefs towards ASD based on their personal beliefs. This might be due to personal beliefs being a reflection of individual perceptions gained from life experiences and training programs, which may lead to logical thinking toward any phenomena.

## 5. Limitations

Despite the importance of the above findings, there some limitations to this study that should be highlighted for future research. The preschool teachers in this study were mostly from normal schools, which might affect the generalizability of the findings of this study. Indeed, the index of beliefs expressed in this study was subjectively informed. There was little evidence to suggest that the beliefs were translated into actions. Therefore, it is difficult to know whether the teachers’ beliefs or attitudes would translate into actual behavior. To avoid giving a rather low overall impression of their belief system, the teachers might have responded with what they perceived as favorable answers. A joint effort to rule out such confounding factors is necessary, with a need for large-scale future studies equipped with standardized training techniques that reach a broader demographic of teachers. Furthermore, the respondents were asked to state their choices in the comment section of the questionnaire. This method might have encouraged them to respond positively, even if they were not sure about a particular item, as explained in other studies [65]. It might have been better to use another method, such as a qualitative approach, by asking the respondents to explain their beliefs concerning the different issues surrounding ASD. Finally, only 5% of the preschool teachers of the sample had had contact with children diagnosed with autism, which erodes negative views. Similar to the interaction hypothesis by Brown et al. [66], several works also proposed that having previous interpersonal contacts with a disabled person would likely reduce negative views. This situation is based on the assumption that one side of the personality is uncovered to the other side, while new understanding emerges as prejudice weakens. Thus, if preschool teachers have had more contact, their understanding would have been more positively affected. Future studies could explore the contact versus non-contact factor, which may have a bearing on the teachers’ understanding of the condition that is unique to ASD children.

## 6. Conclusions

The prevalence of children with ASD now exceeds that of Down’s syndrome, diabetes, or cancer [67]. Hence, preschool teachers should widen their knowledge on ASD to help parents guide their special-needs children. Educated preschool teachers can help parents obtain proper interventions to ensure that their children reach their full potential. To accomplish this end, preschool teachers need to be correctly educated on this disorder, and any inaccurate information among them in particular and in society generally must be discarded. Perhaps a pre-service training among teachers could address these misconceptions and allow easier access for teachers to obtain such important information. Additionally, special-needs education via lectures, workshops, and courses are urgently needed to improve the mindfulness of people working in schools. Mass media can contribute actively by raising awareness regarding ASD knowledge and on how educators can play their roles effectively.

For the first time, this study has highlighted one of the main educational concerns in Yemen. One conclusion that can be drawn is that incorrect beliefs or misunderstandings of the condition that is unique to children with ASD are social problems that transcend specific culture, geography, or society. It concedes to individual countries the right to work hand-in-hand with others to make ASD education compulsory and jointly face the ever-rising ASD misconceptions and in addition, to give these kinds of children complete rights for social justice in education and jobs.

## Figures and Tables

**Table 1:** Sample characteristics.

	*n*	%
**Gender**		
Female	213	100
**Age**		
20–30	57	26.8
31–40	113	53.1
~≥40	43	20.2
**Teaching Experience**		
From 5 and below	54	25.4
Between 5–10	111	52.1
~≥10	48	22.5
**Education**		
High school	24	11.3
Diploma	55	25.8
Bachelor	134	62.9

**Table 2:** Preschool teachers’ beliefs regarding autism spectrum disorder.

	Items	Mean	Std	Rank
	Religion Belief			
1	item1	2.15	0.804	3.00
2	item 2	2.96	0.773	4.00
3	item 3	3.30	1.038	4.00
4	item 4	3.33	0.965	4.00
5	item 5	3.34	0.957	4.00
	Weighted mean	3.024		Moderate
	Std deviation		0.514	
	Social belief	Mean	Std	Rank
6	item 1	3.34	1.064	4.00
7	item 2	3.69	1.185	4.00
8	item 3	3.79	1.273	4.00
9	item 4	3.71	1.295	4.00
10	item 5	3.73	1.373	4.00
11	item 6	3.41	1.466	4.00
12	item 7	3.48	1.503	4.00
13		3.51	1.365	
	Weighted mean	3.64		Low
	Std deviation		0.643	
	Personal belief	Mean	Std	Rank
14	item 1	3.24	1.392	4.00
15	item 2	3.17	1.570	4.00
16	item 3	3.29	1.508	3.00
17	item 4	3.82	1.274	4.00
18	item 5	3.98	1.308	4.00
19	item 6	3.78	1.370	4.00
20	item 7	3.83	1.285	4.00
21	item 8	3.24	1.392	4.00
	Weighted mean	3.62		Low
	Std deviation		0.591	

std: standard deviation.

**Table 3:** ANOVA test between religious belief (DV) and both social and personal belief (IV).

Model		Sum of Squares	df	Mean Square	F	Sig.
1	Regression	120.413	2	60.207	9.880	0.000
	Residual	1279.652	210	6.094		
	Total	1400.066	212			

df: degrees of freedom.

**Table 4:** Coefficients of the structural model assessment.

Model		Unstandardized Coefficients		Standardized Coefficients	t	Sig.
		B	Std. Error	Beta		
1	(Constant)	17.016	1.309		13.001	0.000
	Social	0.142	0.034	284	4.234	0.000
	Personal	0.089	0.042	0.142	2.121	0.035

std: standard deviation.

**Table 5:** Kruskal–Wallis Test (K–W) test for religious belief (age with education levels).

Education Levels	Age	*N*	df	Chi-Square	*p*-Value
High school	20–30	6	2	4.328	0.115
	31–40	1			
	>40	10			
diploma	20–30	17	2	7.536	0.023
	31–40	34			
	>40	11			
Bachelor	20–30	34	2	8.173	0.017
	31–40	78			
	>40	22			
		213			

df: degrees of freedom.

**Table 6:** Kruskal–Wallis Test for religious belief (age with teaching experience).

Teaching Experience	Age	*N*	df	Chi-Square	*p*-Value
From 5 years and below	20–30	12	2	2.875	0.238
	31–40	31			
	˃40	11			
Between 5–10 years	20–30	38	2	2.651	0.266
	31–40	56			
	˃40	17			
Above 10 years	20–30	7	2	14.367	0.001
	31–40	26			
	˃40	15			
		213			

df: degrees of freedom.

**Table 7:** Kruskal–Wallis Test for religious belief (teaching experiences and education levels).

Teaching Experience	Education Levels	*N*	df	Chi-Square	*p*-Value
From 5 years and below	High school	1	2	7.541	0.023
	Diploma	12			
	Bachelor	41			
Between 5–10 years	High School	5	2	16.259	0.000
	Diploma	48			
	Bachelor	58			
Above 10 years	High School	11	2	0.167	0.920
	Diploma	2			
	Bachelor	35			
		213			

df: degrees of freedom.

**Table 8:** Kruskal-Wallis Test for social belief (age with education levels).

Education Levels	Age	*N*	df	Chi-Square	*p*-Value
High School	20–30	6	2	4.077	0.130
	31–40	1			
	˃40	10			
Diploma	20–30	17	2	10.340	0.006
	31–40	34			
	˃40	11			
Bachelor	20–30	34	2	4.315	0.116
	31–40	78			
	˃40	22			
		213			

df: degrees of freedom.

**Table 9:** Kruskal–Wallis Test for social belief (age with teaching experience).

Teaching Experience	Age	*N*	df	Chi-Square	*p*-Value
From 5 years and below	20–30	12	2	1.226	0.542
	31–40	31			
	˃40	11			
Between 5–10 years	20–30	38	2	12.242	0.002
	31–40	56			
	˃40	17			
Above 10 years	20–30	7	2	12.514	0.002
	31–40	26			
	˃40	15			
		213			

df: degrees of freedom.

**Table 10:** Kruskal–Wallis Test for social belief (teaching experiences and education levels).

Teaching Experience	Education Levels	*N*	df	Chi-Square	*p*-Value
From 5 years and below	High school	1	2	2.880	0.237
	Diploma	12			
	Bachelor	41			
Between 5–10 years	High School	5	2	28.786	0.000
	Diploma	48			
	Bachelor	58			
Above 10 years	High School	11	2	10.280	0.006
	Diploma	2			
	Bachelor	35			
		213			

df: degrees of freedom.

**Table 11:** Two-way ANOVA for personal belief.

Source	Type III Sum of Squares	df	Mean Square	F	Sig.
age	42.629	2	21.314	1.665	0.192
educational	160.430	2	80.215	6.264	0.002 *
Teaching experience	3.899	2	1.949	0.152	0.859
age * educational	113.789	3	37.930	2.962	0.033 *
age * experience	156.448	4	39.112	3.054	0.018 *
educational * experience	21.816	3	7.272	0.568	0.637

df: degrees of freedom. F = variation between sample means / variation within the samplest. * Means it is significant. R Squared = 0.312 (Adjusted R Squared = 0.252).
